# Ratchet recruitment in the acute respiratory distress syndrome: lessons from the newborn cry

**DOI:** 10.3389/fphys.2023.1287416

**Published:** 2023-11-01

**Authors:** Gary F. Nieman, Jacob Herrmann, Joshua Satalin, Michaela Kollisch-Singule, Penny L. Andrews, Nader M. Habashi, David G. Tingay, Donald P. Gaver, Jason H. T. Bates, David W. Kaczka

**Affiliations:** ^1^ Department of Surgery, SUNY Upstate Medical Center, Syracuse, NY, United States; ^2^ Roy J. Carver Department of Biomedical Engineering, University of Iowa, Iowa City, IA, United States; ^3^ Department of Medicine, University of Maryland, Baltimore, MD, United States; ^4^ Neonatal Research, Murdoch Children’s Research Institute, Royal Children’s Hospital, Parkville, VIC, Australia; ^5^ Department of Biomedical Engineering, Tulane University, New Orleans, LA, United States; ^6^ Department of Medicine, University of Vermont, Burlington, VT, United States; ^7^ Departments of Anesthesia and Radiology, University of Iowa, Iowa City, IA, United States

**Keywords:** ARDS, airway pressure release ventilation, time-controlled adaptive ventilation, ventilator-induced lung injury, lung recruitment

## Abstract

Patients with acute respiratory distress syndrome (ARDS) have few treatment options other than supportive mechanical ventilation. The mortality associated with ARDS remains unacceptably high, and mechanical ventilation itself has the potential to increase mortality further by unintended ventilator-induced lung injury (VILI). Thus, there is motivation to improve management of ventilation in patients with ARDS. The immediate goal of mechanical ventilation in ARDS should be to prevent atelectrauma resulting from repetitive alveolar collapse and reopening. However, a long-term goal should be to re-open collapsed and edematous regions of the lung and reduce regions of high mechanical stress that lead to regional volutrauma. In this paper, we consider the proposed strategy used by the full-term newborn to open the fluid-filled lung during the initial breaths of life, by ratcheting tissues opened over a series of initial breaths with brief expirations. The newborn’s cry after birth shares key similarities with the Airway Pressure Release Ventilation (APRV) modality, in which the expiratory duration is sufficiently short to minimize end-expiratory derecruitment. Using a simple computational model of the injured lung, we demonstrate that APRV can slowly open even the most recalcitrant alveoli with extended periods of high inspiratory pressure, while reducing alveolar re-collapse with brief expirations. These processes together comprise a ratchet mechanism by which the lung is progressively recruited, similar to the manner in which the newborn lung is aerated during a series of cries, albeit over longer time scales.

## Introduction

Although acute respiratory distress syndrome (ARDS) was identified as a distinct pathologic entity in 1967 (Ashbaugh et al., 1967), there are still no treatments for it other than supportive care involving mechanical ventilation. Unfortunately, mechanical ventilation itself has the potential to increase mortality by causing unintended ventilator-induced lung injury (VILI) (Slutsky and Ranieri, 2013). The current standard of care for ARDS is low tidal volume ventilation (Acute Respiratory Distress Syndrome Network et al., 2000). Nevertheless, mortality associated with ARDS remains unacceptably high (Bellani et al., 2016), which motivates the search for improved approaches to mechanical ventilation.

ARDS typically presents as a heterogeneous pattern of injury on chest imaging, including patches of normal, collapsible, and fully consolidated tissue throughout the lung (Cereda et al., 2016; Cereda et al., 2019). This makes the lung particularly susceptible to further VILI by a mechanism that has been termed the Permeability-Originated Obstruction Response (POOR), manifesting at interfaces between normal and collapsed lung tissue regions (Gaver et al., 2020). The normal parenchyma at such interfaces is distorted by retraction forces exerted by collapsed regions, resulting in elevated tissue stresses that can potentially cause volutrauma. In addition, chronic atelectasis increases the risk of pulmonary infection and fibrosis. However, atelectrauma at these interfaces is also caused by repetitive opening and closing of alveoli with abnormal mechanical properties due to surfactant dysfunction, although such alveoli may not yet fully consolidated (Nieman et al., 2017). While both volutrauma and atelectrauma are key mechanisms of VILI, atelectrauma appears to be the primary driver of its progression (Ramcharran et al., 2022). Both mechanisms together thus cause VILI to spatially propagate outward from consolidated regions in a “POOR-get-POORer” manner, wherein edema-induced surfactant deactivation results in regional areas of instability, with focalized stress multipliers leading to a perpetuating cycle of VILI. This can result in a cascading, irrecoverable injury, which has been termed the “VILI Vortex” (Marini and Gattinoni, 2020; Nieman et al., 2022).

The above considerations indicate that the immediate goal of mechanical ventilation in ARDS should be to prevent unstable alveoli from contributing to atelectrauma. A more long-term goal should be to re-open collapsed and edematous regions and to eliminate the concentrations of high mechanical stress in adjacent aerated tissue that lead to regional volutrauma (Knudsen et al., 2017). Such are the goals of the Open Lung Approach (OLA), which applies positive end-expiratory pressure (PEEP) to prevent alveoli from collapsing during expiration, and periodically administers large, sustained inflations to recruit collapsed tissue (Writing Group for the Alveolar Recruitment for Acute Respiratory Distress Syndrome Trial et al., 2017). While scientific and clinical evidence suggests that the lung would be protected from VILI if these goals could be achieved (Cinnella et al., 2015; Cereda et al., 2016; Gaver et al., 2020), the OLA has failed to reduce the mortality associated with ARDS in randomized clinical trials (Writing Group for the Alveolar Recruitment for Acute Respiratory Distress Syndrome Trial et al., 2017). Moreover, physiologic, and radiographic indices (Writing Group for the Alveolar Recruitment for Acute Respiratory Distress Syndrome Trial et al., 2017) suggest that the OLA, as currently practiced, does not maintain durable recruitment of the unstable regions of the injured lung. Indeed, transient recruitment of the lung may increase the number of alveoli subject to atelectrauma, if subsequent derecruitment is not prevented (Halter et al., 2003).

The question remains*: is there an approach to mechanical ventilation that safely reduces repetitive atelectrauma, while simultaneously reopening collapsed and edematous lung tissue*? In this paper, we consider the strategy used by the full-term newborn to open the fluid-filled lung during the initial breaths of life. The newborn’s cry after birth shares key similarities with the Airway Pressure Release Ventilation (APRV) modality, in which the expiration duration is sufficiently short to minimize end-expiratory derecruitment. Using a simple computational model of the injured lung that incorporates mechanisms for fast and slow recruitment, we demonstrate that APRV, when administered in an appropriate manner, may be an answer to the above question.

## Opening the fluid-filled lung of the newborn

At birth, the healthy, full-term infant faces the same problem as the clinician managing a patient with ARDS—that is, how to open a fluid-filled lung. The ability to maintain aeration in previously obstructed, flooded or collapsed lung tissue is critical to the survival of both the newborn and the patient with ARDS. In a recent paper, Tingay et al. (2021) explored a strategy by which nature opens and maintains recruitment in the newborn lung.

The lungs of an early to full-term newborn (i.e., ≥36 weeks gestation) with a functioning surfactant system are filled with amniotic fluid at birth. Upon delivery, the newborn inhales vigorously, and often emits a cry with its first exhalation. This initial inspiration forces air into the small airways and acini, displacing amniotic fluid into more distal lung regions, where it may be cleared through the interstitium (Jain and Eaton, 2006). Following initial inhalation, the physiological problem shifts to preventing newly aerated tissue from collapsing during the subsequent exhalation. To do so, the neonate is believed to impede expiratory flow by partially closing the glottis to reduce expiratory flow, which transiently raises pressure in the lung and helps to move gas distally into partially flooded alveoli. A subsequent series of cries continues to ratchet the lung progressively open, until it is fully recruited and surfactant function is established. At this point, the alveoli remain open and stable, such that normal tidal ventilation can be maintained without further derecruitment. Thus, the first ratcheting breaths of the crying newborn adds gas volume to the portion of open lung with each inspiration, without losing ground during each expiration.

## Airway pressure release ventilation for patients with ARDS

Edematous and collapsed regions of the lung in ARDS can be difficult to recruit because of proteinaceous fluid entering alveoli through the disrupted alveolar-capillary barrier, which deactivates pulmonary surfactant. Even small amounts of recruitment achieved through the application of high airway pressure may be lost quickly once such pressure is released (Lu et al., 2006). This is due to both time and pressure dependencies of alveolar recruitment and derecruitment. In other words, high airway pressures do not necessarily open collapsed alveoli immediately. Recruitment may require a long time to manifest when surfactant deactivation is severe—possibly even longer than the duration of even the most protracted single recruitment maneuver.

The APRV modality may allow for such gradual recruitment, provided it is administered with sufficiently short expiratory durations. With APRV, expiratory durations are short enough such that recruited alveoli are not given sufficient time to close. This strategy, which reopens edematous lungs (Veneroni et al., 2020; Tingay et al., 2021), also gradually ratchets open the injured lung open over an extended period of hours or days, during which adequate ventilation may be achieved via rapid and periodic removal of alveolar gas This is similar to the application of an “adaptive” PEEP to the lungs (Lam et al., 2020; Herrmann et al., 2021). A more personalized approach to APRV, termed the Time-Controlled Adaptive Ventilation (TCAV) (Nieman et al., 2020), has been shown to be an effective means of choosing expiratory durations that balance alveolar CO_2_ elimination vs. alveolar re-collapse. TCAV adjusts the expiratory duration according to the slope of the expiratory flow curve, which is governed by the emptying time-constant of the respiratory system (Herrmann et al., 2021). With TCAV, expiration is terminated when the magnitude of expiratory flow has fallen to 75% of its peak value at the start of expiration. TCAV has been shown to be highly protective against VILI in clinically relevant animal models of ARDS (Roy et al., 2013; Kollisch-Singule et al., 2015).

## The newborn cry compared to APRV: a computational modeling analysis

**FIGURE 1:**
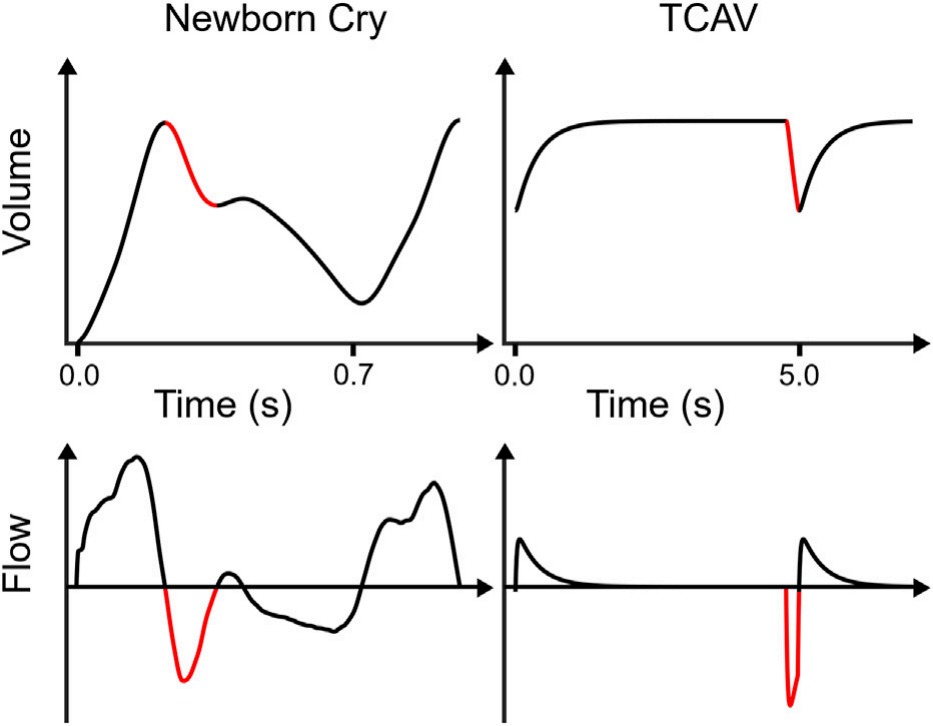
Comparisons between newborn cry and TCAV lung volume vs. time and respiratory flow vs. time. The newborn lung volume waveform shows data from a surrogate measurement (i.e., relative volume change since birth assessed by electrical impedance tomography) published by Tingay et al. (2021), then smoothed and time-differentiated to obtain flow. The TCAV waveforms were simulated in a single-compartment resistance-compliance-inertance model of respiratory mechanics. TCAV applies a prolonged high airway pressure, maintaining high lung volume. Both waveforms exhibit a rapid initial exhalation (red) with large negative flow rates for a brief time before an expiratory “brake” abruptly halts volume change. However, the newborn waveform continues with a second, slower exhalation phase, whereas TCAV immediately reinflates. Note the increase in the newborn lung volume between the beginning and end of the first breath. Note that the newborn breath duration is approximately 0.7 s, whereas each simulated TCAV cycle is 5 s.

Figure 1 compares the volume vs. time and flow vs. time relationships for a newborn cry and TCAV that demonstrate some common features. They both employ a rapid inspiratory phase to aerate lung tissue. The newborn follows this inspiration with a rapid exhalation, that is, then impeded through mechanisms such as glottal restriction, which leads to a prolongation exhalation. For TCAV, the breath-hold maneuver precedes exhalation but, similar to that of the newborn, this exhalation is quickly interrupted to prevent collapse of the newly recruited tissue. Such a breath profile leads to the question: “*Can the collapsed and edematous lung with ARDS also be ratcheted opened, similar to the neonatal lung?*” A secondary question is: “*Can such ratcheting occur simultaneously with the maintenance of adequate ventilation of alveoli that are already open?*”

To answer these questions, it is important to understand how quickly the expiration should be terminated. This is a critical issue because derecruitment in the injured lung can be rapid, so significant alveolar closure can occur even when expiration is substantially shorter than in conventional mechanical ventilation. If derecruitment occurs, alveoli that close must be forced open during the next inspiration, resulting in atelectrauma. This may be a highly injurious process that quickly leads to severe VILI if allowed to occur repeatedly (Kollisch-Singule et al., 2014).

**FIGURE 2:**
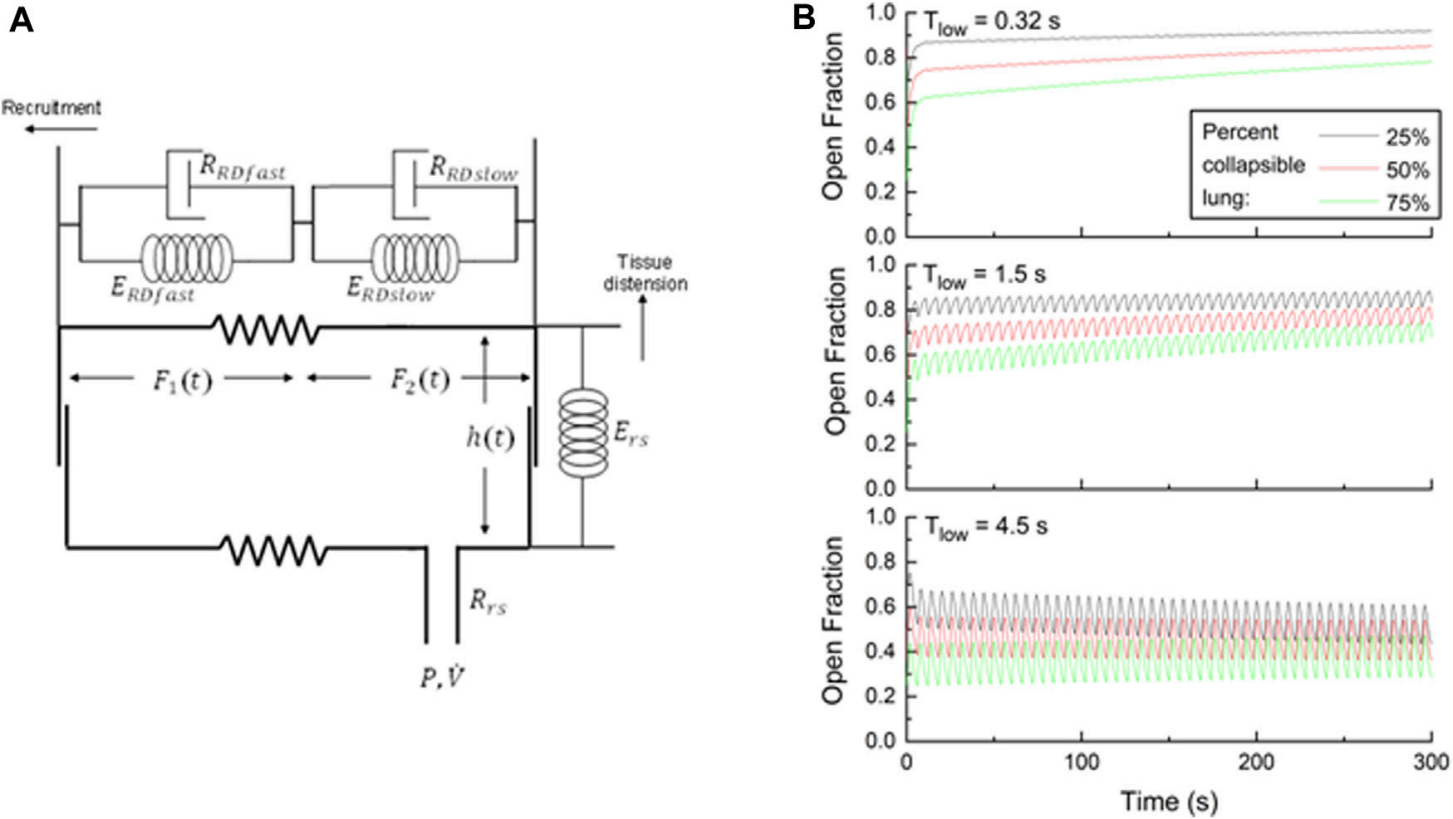
**(A)** Single-compartment model of the respiratory system in which the alveolar compartment expands vertically to represent tissue distension, but horizontally to represent recruitment. Variables for the model parameters are defined in the text. **(B)** Open fractions of the lung $F\left(t\right)$ , simulated by the computational model depicted in **(A)**. The model was mechanically ventilated with APRV using a breath period of 6 s, starting with 25% (black), 50% (red), and 75% (green) derecruitment. Simulations were performed with three different expiratory durations (*T*_*low*_) as indicated.

In a previous study (Bates et al., 2020), we represented the lung as a single alveolar compartment that can expand in two orthogonal directions, as illustrated in Figure 2A. Airway pressure and flow are denoted as *P* and $\dot{V}$ , respectively. Vertical expansion corresponds to distension of the open lung, measured by $h\left(t\right)$ , while horizontal expansion corresponds to an increase in the open lung fraction (i.e., recruitment of closed lung units), measured by $F\left(t\right)$ . It should be noted that vertical and horizontal directions are not related to the physical orientation of the lung deformation; rather, they are used for illustrative purposes. The intrinsic elastic properties of the respiratory tissues are represented by a spring with stiffness $E_{rs}$ , corresponding to the elastance of the respiratory system when the lung is fully recruited. As the lung derecruits, represented by a decrease in the lateral dimension of the alveolar unit in the model, the apparent respiratory system elastance increases above the value of $E_{rs}$ , in inverse proportion to the fraction of lung that remains open. The alveolar compartment is served by a conduit representing respiratory system resistance $R_{rs}$ . We assume that *R*_*rs*_ is dominated by the flow resistances of the endotracheal tube and ventilator circuit, which are not affected by recruitment and derecruitment. Thus, for our simulations, $R_{rs}$ remains fixed regardless of the state of the lung. The time-constant of the respiratory system that governs how rapidly the lung empties during passive expiration is given by τ _rs_ = $R_{rs}{/E}_{rs}$ . Accordingly, the time required for lung deflation decreases as $E_{rs}$ increases due to derecruitment and the effectively smaller lung volume. The rate at which the model recruits and derecruits are determined by two horizontal spring-and-dashpot combinations, which account for rapid (i.e., intratidal) and slow (i.e., minutes to hours) changes in the amount of opened lung. The rapid changes are governed by the two parameters $E_{RDfast}$ and $R_{RDfast}$ that together determine a time-constant $\tau_{RDfast}=R_{RDfast}/E_{RDfast}$ of fast recruitment and derecruitment. The corresponding slow changes are determined by $\tau_{RDslow}=R_{RDslow}/E_{RDslow}$ . Additional technical details of the model are presented in the Online Supplement.

Figure 2B shows how the fraction of open lung $F\left(t\right)$ changes over time when the model simulation is initiated with 25%, 50% and 75% of the lung recruited (i.e., equally distributed to the fast and slow compartments in each case), and is then subjected for 300 s (i.e., 5 min) to 50 breaths of APRV. The APRV parameters for this simulation consisted of an inspiratory pressure (*P*_*high*_) of 30 cm H_2_O, a release pressure (*P*_*low*_) of 0 cm H_2_O, an inspiratory duration (*T*_*high*_) of 5.68 s, and an expiratory duration (*T*_*low*_) of 0.32 s. Using this value of *T*_*low*_ for the 50% derecruitment simulation resulted in the first expiration being terminated when the magnitude of expiratory flow had fallen to approximately 75% of its peak value. The corresponding end-expiratory flows were 71% and 79%, respectively, for the 75% and 25% derecruitment simulations. In other words, *T*_*low*_ = 0.32 s *approximated* the TCAV strategy in each case. Minimal breath-to-breath variations occur in $F\left(t\right)$ as a result of the brevity of expiration, although the long-term trend is for $F\left(t\right)$ to increase progressively as the lung is slowly ratcheted opened under the sustained influence of the high inspiratory pressure. These simulations also demonstrate that when a greater proportion of the lung is initially derecruited, the subsequent rate of recruitment is increased, which speaks to the efficacy of TCAV for gradually reopening the collapsed lung.

Repeating this simulation with *T*_*high*_ = 4.5 s and *T*_*low*_ = 1.5 s illustrates the problem associated with using typical APRV settings without the TCAV strategy. The long-term recruitment trends remain, albeit delayed relative to the simulations with *T*_*low*_ = 0.32 s, but there are now noticeable oscillations in $F\left(t\right)$ resulting from intratidal recruitment and derecruitment. When the simulations are repeated yet again, this time with *T*_*high*_ = 1.5 s and *T*_*low*_ = 4.5 s (i.e., shorter inspiratory duration but long expiration duration), similar to the timing of conventional mechanical ventilation, the intratidal oscillations in $F\left(t\right)$ are substantial, portending a severe degree of atelectrauma. Accordingly, these model simulations recapitulate injurious phenomena at play in the lung during mechanical ventilation, and demonstrates the key principles motivating the use of APRV with the TCAV strategy in ARDS.

## Conclusion

In summary, we have reviewed the key similarities and differences between the newborn cry and the mechanical breath of the APRV/TCAV modality. Nature has devised a unique mechanical strategy for opening the newborn lung, allowing it to transition rapidly from its fluid-filled state *in utero* to the air-filled state necessary for survival outside the womb. The newborn lung achieves this vital function by ratcheting lung tissue open over a series of initial breaths that incorporate brief expirations, to avoid undoing the gains made with each previous inspiration. A similar challenge faces the clinician who must ventilation the collapsed and edematous lung with ARDS, but with an added complication. Recruitment in an injured lung typically takes longer to manifest than in the neonatal lung, with ongoing ventilation of those alveoli already opened. APRV administered using TCAV achieves these goals, with rapid lung inflation to move fluid from the airways into the interstitial spaces, thus opening collapsed airways and alveoli. Extended periods of high inspiratory pressure slowly recruits even the most recalcitrant alveoli, while brief expirations avoid re-collapse of those alveoli already opened. These processes together comprise a ratchet mechanism by which the lung is progressively recruited, much in the same way that the newborn lung is aerated during a series of cries, albeit over a longer time scale.

## Data Availability

The raw data supporting the conclusion of this article will be made available by the authors, without undue reservation.
