# Supplementary material for: Ratchet recruitment in the acute respiratory distress syndrome: lessons from the newborn cry
Source: Front Physiol. 2023 Nov 1;14:1287416. doi: 10.3389/fphys.2023.1287416 (PMC10646689; doi:10.3389/fphys.2023.1287416)
Supplement: Supplementary file 1 [file DataSheet1.PDF]

ONLINE DATA SUPPLEMENT

Ratchet Recruitment in the Acute Respiratory Distress Syndrome: Lessons from the Newborn Cry

Gary F. Nieman[1], Jacob Herrmann[2], Joshua Satalin[1], Michaela Kollisch-Singule[1], Penny L. Andrews[3], Nader M. Habashi[3], David G. Tingay[4], Donald P. Gaver[5], Jason H.T. Bates[6], David W. Kaczka[2,7]

[1]Department of Surgery
SUNY Upstate Medical Center, Syracuse, NY, USA

[2]Roy J. Carver Department of Biomedical Engineering
University of Iowa, Iowa City, IA, USA

[3]Department of Medicine
University of Maryland, Baltimore, MD, USA

[4]Neonatal Research, Murdoch Children's Research Institute, Royal Children's Hospital, Flemington Road, Victoria, Australia

[5]Department of Biomedical Engineering
Tulane University, New Orleans, LA, USA

[6]Department of Medicine
University of Vermont, Burlington, VT, USA

[7]Departments of Anesthesia and Radiology
University of Iowa, Iowa City, IA, USA

Based on the model of Figure 2-A, if alveolar pressure (P_A) is held constant, the fraction of open lung will eventually converge toward a steady-state value determined by the combined stiffness of the two horizontal springs. This combined stiffness is

$$E_{RD} = \frac{E_{RDslow}E_{RDfast}}{E_{RDslow} + E_{RDfast}} \quad \text{(E1)}$$

We assume that E_{RDslow} and E_{RDfast} are equal, in which case $E_{RDslow} = E_{RDsfast} = 2E_{RD}$. The two horizontal dashpots in Figure 2-A prevent the steady-state open fraction from being attained immediately following a change in P_A, and represent the resistance to opening or closing of lung units provided by the motion of a plug of airway fluid or to fluid flows in the airway lining liquid (Fujioka et al., 2008). Instead, the steady-state value is approached asymptotically as the dashpots slide under the force exerted by the spring. The two Maxwell elements together thus imbue the model with dynamic recruitment/derecruitment behavior whereby the fraction of open lung at any point in time is determined by prior excursions in P_A in addition to its current value.

At any point in time (t) the parenchymal tissue is assumed to comprise two populations of lung units – those that are open and distended by P_A, and those that are closed. A critical opening/closing pressure (P_{crit}) is associated with each unit. At steady state (i.e., when a given P_A is maintained indefinitely) those units for which for $P_A > P_{crit}$ will be open and those for which $P_A \leq P_{crit}$ will be closed. For simplicity, we assume that the critical opening and closing pressures for a given unit are the same, so there is only one value of P_{crit} for each unit regardless of whether it is subject to recruitment or derecruitment (Amini et al., 2017).

Following our previous work and that of others (Massa et al., 2008), we assume that P_{crit} is a Gaussian function of P_A, with mean μ and standard deviation σ. This means that the steady-state open fraction, F_{stat}, is the cumulative Gaussian function

$$F_{stat}(P_A) = \frac{1}{\sqrt{2\pi\sigma^2}} \int_{-\infty}^{P_A} e^{-\frac{(P-\mu)^2}{2\sigma^2}} dP \quad \text{(E2)}$$

Computational model fitting to experimental data in mice suggests that μ increases monotonically with surface tension at the air-liquid interface (i.e., with the increasing surfactant dysfunction that accompanies VILI), while σ remains fixed (Massa et al., 2008). Accordingly, we assume that the progression of VILI is defined entirely by the function $\mu(t)$.

The consequence of P_A acting on the intrinsic tissue elastance of the respiratory system, E_{rs}, is given by

$$P_A(t) = h(t)E_{rs} \quad \text{(E3)}$$

while the volume of the lung, $V(t)$, is given by

$$V(t) = h(t)[F_1(t) + F_2(t)] \quad \text{(E4)}$$

Where $F_1(t)$ and $F_2(t)$ are the fractions of open lung represented by the extensions of springs E_{RDfast} and E_{RDslow}, respectively. E_{RD} (Eq. E1) is thus a nonlinear function defined by the ratio of P_A to F_{stat} as expressed in Eq. E2. Furthermore, because $E_{RDfast} = E_{RDslow}$, we let the equilibrium values of $F_1(t)$ and $F_2(t)$ each be $F_{stat}/2$.

Changes in F_1 and F_2 do not take place as soon as P_A changes; rather, the lung opens transiently when pressures are suddenly raised, and closes transiently when pressures are suddenly lowered. The dynamics of these transients are assumed to be of first order. That is, if the lung is held at a fixed value of P_A, F approaches $F_{stat}(P_A)$ asymptotically at a rate that depends only on the difference between $F_1 + F_2$ and $F_{stat}(P_A)$. Individually, F_1 and F_2 each approach $F_{stat}(P_A)/2$. These dynamics are created in the model by having the dashpot resistances R_{RDfast} and R_{RDslow} be fixed fractions of E_{RDfast} and E_{RDslow}, respectively. These fractions are chosen such that τ_{RDfast} is in the order of a second while τ_{RDslo} is much greater than this. This is motivated by

experimental derecruitability tests showing that, following a recruitment maneuver, the rapid decruitment of the lung at a fixed inflation pressure is quasi-exponential (Albert et al., 2009). These dynamics are thus governed by the equation

$$\dot{F}_1(t) = \frac{1}{\tau_{RDfast}}\left[\frac{F_{stat}}{2}\big(P_A(t)\big) - F_1(t)\right] \tag{E5}$$

and

$$\dot{F}_2(t) = \frac{1}{\tau_{RDslow}}\left[\frac{F_{stat}}{2}\big(P_A(t)\big) - F_2(t)\right] \tag{E6}$$

The simulation of pressure-controlled mechanical ventilation requires a parameter corresponding to respiratory resistance, R_{rs}, so that the lung empties with a realistic time-constant (R_{rs} includes the flow resistance of the endotracheal tube and expiratory limb of the ventilator). If the ventilator applies an airway opening pressure waveform $P_{ao}(t)$ to the trachea, the flow, $\dot{V}(t)$, into the lungs is

$$\dot{V}(t) = \frac{P_{ao}(t) - P_A(t)}{R_{rs}} = h(t)\dot{F}(t) + \dot{h}(t)F(t) \tag{E7}$$

which gives

$$\dot{h}(t) = \frac{\dot{V}(t) - h(t)\dot{F}(t)}{F(t)} \tag{E8}$$

For a lung in a fixed state of injury, the behavior of this model is governed by 6 free parameters - E_{rs}, R_{rs}, σ, μ, τ_{RDfast}, and τ_{RDslow}. Equations E1 to E8 define how the model behaves in response to any prescribed ventilator pressure or volume waveform. First, we integrated these equations at 100 Hz with the initial condition $F_1(t) = F_2(t) = 0.25$ (i.e., the simulation began at 50% lung recruitment). The values of the other parameters were $E_{rs} = 10$ cmH$_2$O.L^{-1}, $R_{rs} = 15$ cmH$_2$O.s.L^{-}

[1], $\sigma = 6$ cmH_2O, $\mu = 6$ cmH_2O, $\tau_{RDfast} = 2$ s, and $\tau_{RDslow} = 500$ s. The APRV pressure parameters for this simulation were P_{high} = 30 cmH_2O and P_{low} = 0 cmH_2O.

We performed model simulations with three different degrees of initial lung derecruitment – 25%, 50% and 75%. In each case, we used an inspiratory duration (T_{high}) of 5.68 s, and an expiratory duration (T_{low}) of 0.32 s. Using this value of T_{low} for the 50% derecruitment simulation resulted in the first expiration being terminated when the magnitude of expiratory flow had fallen to approximately 75% of its peak value. The corresponding end-expiratory flows were 71% and 79%, respectively, for the 75% and 25% derecruitment simulations. In other words, T_{low} = 0.32 s approximated the TCAV strategy in each case. Minimal breath-to-breath variations occur in $F(t)$ as a result of the brevity of expiration, although the long-term trend is for $F(t)$ to increase progressively as the lung is slowly ratcheted opened under the sustained influence of the high inspiratory pressure. These simulations also demonstrate that when a greater proportion of the lung is initially derecruited, the subsequent rate of recruitment is increased, which speaks to the efficacy of the TCAV strategy for gradually reopening the collapsed lung.

Repeating these simulations with T_{high} = 4.5 s and T_{low} = 1.5 s illustrates the problem associated with using typical APRV settings without the TCAV strategy. The long-term recruitment trends remain, albeit delayed relative to the simulations with T_{low} = 0.32 s, but there are now noticeable oscillations in $F(t)$ resulting from intratidal recruitment and derecruitment. When the simulations are repeated yet again, this time with T_{high} = 1.5 s and T_{low} = 4.5 s, the breath-by-breath oscillations in $F(t)$ are greatly accentuated and the trend towards long-term recruitment is lost.

References

ALBERT, S. P., DIROCCO, J., ALLEN, G. B., BATES, J. H., LAFOLLETTE, R., KUBIAK, B. D., FISCHER, J., MARONEY, S. & NIEMAN, G. F. 2009. The role of time and pressure on alveolar recruitment. *J Appl Physiol (1985),* 106**,** 757-65.

AMINI, R., HERRMANN, J. & KACZKA, D. W. 2017. Intratidal overdistention and derecruitment in the injured lung: a simulation study. *IEEE Trans Biomed Eng,* 64**,** 681-689.

FUJIOKA, H., TAKAYAMA, S. & GROTBERG, J. B. 2008. Unsteady propagation of a liquid plug in a liquid-lined straight tube. *Phys Fluids (1994),* 20**,** 62104.

MASSA, C. B., ALLEN, G. B. & BATES, J. H. 2008. Modeling the dynamics of recruitment and derecruitment in mice with acute lung injury. *J Appl Physiol (1985),* 105**,** 1813-21.
